# Impromptu Response to Adverse Weather: One School’s Successful Application of Digital Interviewing for Medical School Admissions

**DOI:** 10.15694/mep.2017.000034

**Published:** 2017-02-21

**Authors:** Linda S. Nield, Alyson S. Carpenter, Leigh A. Pratt, Manuel Vallejo, Mark Paternostro, Norman D. Ferrari III

**Affiliations:** 1 West Virginia University School of Medicine

**Keywords:** medical school admissions, interviews, digital interviews, recruitment

## Abstract

This article was migrated. The article was marked as recommended.

In-person interviewing is the norm during the medical school application process, but it is not always possible due to reasons such as exorbitant cost of travel, work or residence outside the country and adverse travel conditions. In January 2016 during Winter Storm Jonas in the eastern United States of America, officials at the West Virginia University School of Medicine (WVU SOM) mandated a closure of the campus resulting in the cancellation of twenty on-site medical school interviews. The impromptu solution to overcoming this obstacle to in-person interviewing is described here as a case study. The successful use of digital interviewing has already been described at the residency and fellowship level; we believe this is the first reported case study about the successful use of FaceTime interviewing at the medical school level, and it may encourage admissions officials to expand the use of this modality in medical student recruitment.

## Introduction

In-person interviewing is the norm during the medical school application process, but it is not always possible due to reasons such as exorbitant cost of travel, work or residence outside the country and adverse travel conditions. In January 2016 during Winter Storm Jonas in the eastern United States of America, officials at the West Virginia University School of Medicine (WVU SOM) mandated a closure of the campus on a Friday and the following Monday resulting in the cancellation of twenty on-site medical school interviews. Although five candidates had already arrived in town and were situated in a hotel, on-campus interviews were not possible. The impromptu solution to overcoming this obstacle to in-person interviewing is described here as a case study.

## Case Study

In response to a campus closure, twenty medical school applicants were offered a FaceTime interview in lieu of an in-person interview during Winter Storm Jonas in January of 2016. Applicants agreeable to a FaceTime interview received emails with links to information and video presentations about the WVU SOM and its three campuses. The FaceTime interviews were conducted separately one-on-one by the Admissions Dean and the Chairman of the Committee on Admissions (COA), lasting approximately 45 minutes each. Since the inclement weather prohibited travel, the interviewers conducted the interviews in their respective homes, while the interviewees remained at their own locations in their homes or hotel rooms. The interviewers thoroughly reviewed the applicants’ files beforehand and conducted their interviews in a similar style to a typical in-person interview. Unlike in-person applicants, FaceTime applicants did not have the opportunity to interact with other COA members, student services personnel or current medical students, and they were unable to have an actual facility tour. Post-interview evaluation forms of the candidates, identical to those forms for in-person interviews, were completed by the interviewers. The candidates were subsequently presented to the COA in identical fashion to those candidates interviewed in-person. Similar to candidates interviewed in -person, the FaceTime candidates completed a post-interview survey about their interview experience.

Nine of the twenty applicants (45%) chose to participate in the FaceTime interview in lieu of the standard in-person interview. Five (56%) of the applicants were situated in a hotel, and four (44%) were at home. No technical difficulties occurred, except for slight lag in message transmission during one interview. The rankings by the COA of these nine applicants were as follows: 44% were accepted and 56% were waitlisted for the Class of 2020. Ultimately, 33% of the applicants that interviewed via FaceTime matriculated. In comparison, for the 11 applicants who refused a FaceTime interview, 18% withdrew their application, 9% were accepted, 55% were waitlisted, and 18% were rejected. Ultimately, none of the 11 students matriculated. In regards to the entire applicant pool of individuals who interviewed in-person (93% of those offered an in-person interview), the rankings by the COA were as follows: 21% were accepted, 63% were waitlisted, 12% ultimately matriculated, and 17% were rejected. In comparing the three groups of the FaceTime candidates (N=9), the candidates who chose not to use FaceTime (N=11), and those applicants who interviewed in-person through the rest of the interview process (N=872), no significant differences were noted with respect to acceptance rates, waitlisted rates and rejection rates. There was a trend (p=0.07) favoring those who used FaceTime to eventual matriculation.

Results of the post-interview survey by the FaceTime interviewees revealed that 100% agreed that the interviewers thoroughly reviewed their applications, 100% described the interview as “just right,” and 86% rated the over-all experience at “excellent,” while 14% rated the over-all experience as “fair.” In regards to the effectiveness of the digital interview in comparison to other in-person interviews experienced by the interviewees, 71% rated the FaceTime interview as “just as effective,” while 29% rated it as “not as effective.” Fifty-seven percent felt less stress for the FaceTime vs. the in-person interview.

## Discussion

Although medical schools already use digital interviews for recruitment in special circumstances, the literature is scarce concerning the effectiveness of these interviews. The experience at the WVU SOM provides evidence that FaceTime interviews are a viable alternative to in-person interviews when obstacles and unforeseeable circumstances (like severe weather) preclude campus visits by applicants. The use of FaceTime for interviews in lieu of an in-person interview was considered effective for both the applicants and our institution. As digital interviewing is becoming more widely used in the general workforce, resources on the topic are becoming available ( Bailo, 2014; Higgins, 2014). Travel and accommodation costs ( AAMC, 2016) adding to increasing debt, inclement weather and residence in another country are sample reasons for medical schools to consider offering FaceTime interviews to certain applicants who experience these obstacles. Winter Storm Jonas was the impetus for the use of FaceTime interviews at the WVU SOM, and the interview process was successful for medical school recruiting. Although not previously reported for medical school recruiting, the use of digital interviewing has already been reported to be successful in the recruiting of residents and/or fellows in the fields of Pediatrics, Family Medicine, Anesthesiology, Urology and Gastroenterology ( Nield et al, 2013; Edje et al, 2013; Vadi et al, 2016; Shah et al, 2012; Daram et al, 2014). We believe this is the first case study report about the successful use of FaceTime interviewing at the medical school level, and it may encourage admissions officials to expand the use of this modality in recruitment.

## Take Home Messages

• The case study by West Virginia University School of Medicine provides evidence that digital interviewing can be a successful modality for student recruitment.
• Although digital interviewing has been employed in just special circumstances, medical school admissions officials should consider its use more routinely as a means to decrease travel and accomodation costs for applicants, with the ultimate goal of potentially decreasing overall student debt.

## Notes On Contributors

Linda S. Nield, MD is the Assistant Dean for Admissions and Professor in the Departments of Medical Education and Pediatrics at West Virginia University School of Medicine.

Alyson S. Carpenter, BA is the Admissions Analyst in the Department of Medical Education at West Virginia University School of Medicine.

Leigh A. Pratt, MS is the Admission Coordinator in the Department of Medical Education at West Virginia University School of Medicine.

Manuel Vallejo, MD, DMD is the Assistant Dean and Designated Institutional Official for Graduate Medical Education and Professor in the Departments of Medical Education and Anesthesia at West Virginia University School of Medicine.

Mark Paternostro, PhD is the former Chair of the Committee on Admissions, and Associate Professor and Associate Chair for Education for the Department of Physiology and Pharmacology at West Virginia University School of Medicine.

Norman D. Ferrari III, MD is the Vice Dean for Education and Academic Affairs, and Professor and Chair of the Department of Medical Education at West Virginia University School of Medicine.
