# Supplementary material for: Impromptu Response to Adverse Weather: One School’s Successful Application of Digital Interviewing for Medical School Admissions
Source: MedEdPublish (2016). 2017 Feb 21;6:34. [Version 1] doi: 10.15694/mep.2017.000034 (PMC10885236; doi:10.15694/mep.2017.000034)
Supplement: Peer review history

# Reviewer report: Reviewer response for version 1

**DOI:** 10.21956/mep.19447.r28628

**Published:** 2018-05-11

**Authors:** Anthony David.M

**Affiliations:** 1 Soho

This review has been migrated. The reviewer awarded 5 stars out of 5 This is an innovative and well written paper. Congratulations to the authors. I have had some experience in taking interviews via Skype for faculty positions. I find that it is a good learning exercise. Sometimes the connections are poor and can cause disruptions but most of the time it is fine. The amount of time, money and effort saved by these medical school aspirants is enormous. Perhaps it is time to consider whether Skype/Facetime interviews can replace live interviews in many situations!

Reviewer Expertise:

NA

No decision status is available

# Reviewer report: Reviewer response for version 1

**DOI:** 10.21956/mep.19447.r28630

**Published:** 2017-03-07

**Authors:** Michelle McLean

**Affiliations:** 1 Bond University

This review has been migrated. The reviewer awarded 4 stars out of 5 Thanks, Nield et al. for reminding us that technology can assist us. Having just lived through five days of MMIs with more than 250 students from across Australia and NZ, with many early mornings and evenings, surely there must be a more cost-effective way? Not only are there costs to the university but students and their parents must travel and perhaps overnight near the university. The challenge will be organizing eight MMI stations remotely vs. one long interview.

Reviewer Expertise:

NA

No decision status is available

# Reviewer report: Reviewer response for version 1

**DOI:** 10.21956/mep.19447.r28629

**Published:** 2017-02-21

**Authors:** Trevor Gibbs

**Affiliations:** 1 AMEE

This review has been migrated. The reviewer awarded 4 stars out of 5 After running the various "Medical Education in Difficult Circumstances" activities at last year's AMEE Conference in Barcelona, it was interesting to hear of the various "difficult circumstance". For many years these focused on resource and financial issues, but now that spectrum is much wider and this paper highlights one of those varied"difficult circumstances".This paper was very clearly written and introduced a somewhat novel approach to interviewing medical students when face-to-face interviewing was not possible. I felt that the results were fascinating and showed that there was little difference from a face-to-face interview.If I was going to be critical at all about the paper I wondered if a more qualitative approach might have been more effective in understanding some of the reasons that some students passed and others failed to matriculate. I would also have liked to have heard from the interviewers as to their perceptions of the activity.Despite these reservations, I feel that this is a useful paper and I am sure we are going to hear more about these types of interviews in future years

Reviewer Expertise:

NA

No decision status is available
